# Age and hippocampal volume predict distinct parts of default mode network activity

**DOI:** 10.1038/s41598-019-52488-9

**Published:** 2019-11-05

**Authors:** Matteo De Marco, Sebastien Ourselin, Annalena Venneri

**Affiliations:** 1 Department of Neuroscience, Medical School, University of Sheffield, Royal Hallamshire Hospital, Beech Hill Road, S10 2RX Sheffield, UK; 2 Department of Imaging and Biomedical Engineering, King’s College London, Strand, London UK

**Keywords:** Neural ageing, Diagnostic markers

## Abstract

Group comparison studies have established that activity in the posterior part of the default-mode network (DMN) is down-regulated by both normal ageing and Alzheimer’s disease (AD). In this study linear regression models were used to disentangle distinctive DMN activity patterns that are more profoundly associated with either normal ageing or a structural marker of neurodegeneration. 312 datasets inclusive of healthy adults and patients were analysed. Days of life at scan (DOL) and hippocampal volume were used as predictors. Group comparisons confirmed a significant association between functional connectivity in the posterior cingulate/retrosplenial cortex and precuneus and both ageing and AD. Fully-corrected regression models revealed that DOL significantly predicted DMN strength in these regions. No such effect, however, was predicted by hippocampal volume. A significant positive association was found between hippocampal volumes and DMN connectivity in the right temporo-parietal junction (TPJ). These results indicate that postero-medial DMN down-regulation may not be specific to neurodegenerative processes but may be more an indication of brain vulnerability to degeneration. The DMN-TPJ disconnection is instead linked to the volumetric properties of the hippocampus, may reflect early-stage regional accumulation of pathology and might be of aid in the clinical detection of abnormal ageing.

## Introduction

The default-mode network (DMN) is a neural pathway that de-activates during overt cognitive processing and activates when one engages in internally-driven mental operations, i.e., conceptual-semantic and “self-projecting” processing like envisioning the future or autobiographical remembering^1,2^. The main set of areas where intrinsic DMN functional connectivity is observed includes the precuneus^3^, the posterior cingulate/retrosplenial complex, inferior parietal lobule, medial-prefrontal cortex, lateral temporal cortex and the hippocampal formation^4^.

A body of studies has highlighted that both normal ageing and Alzheimer’s disease (AD) entail significant down-regulation of the DMN. Gradual down-regulation in DMN functional connectivity is observed from young to late adulthood, and the postero-medial part of the network, including the posterior cingulate/retrosplenial cortex and the precuneus, is one of the regions most influenced by advancing age^5,6^. This holds valid also after accounting for age-associated volumetric decreases^7^. Reduction of connectivity in the posterior portion of the DMN is also observed in healthy adults with amyloid-β burden^8^, young carriers of *PSEN1*/*PSEN2*/*APP* mutations^9^, patients with a clinical diagnosis of AD^10–12^, adults with a diagnosis of amnestic MCI^13,14^, and in the continuum from healthy adulthood, to MCI, to AD dementia^15,16^.

These findings indicate that the links between the DMN and ageing and between the DMN and AD may be qualitatively not specific. Although the effect of ageing and AD on the posterior part of DMN is qualitatively similar, however, this appears exacerbated in AD^17^.

The studies that have investigated the effects of ageing and AD on the DMN were devised based on the statistical comparison (i.e., via a between-sample *t* test) of patients and age-matched healthy adults^11,17,18^. This inferential method centres around group membership as a unique dichotomic independent variable. With this design it is assumed that each measurement can contribute to one group only. At present, however, it is unknown to what extent reduced DMN functional connectivity seen in a single patient is the result of AD, ageing, and/or their interplay. On one hand, no patient with AD is immune to the incidental effects of ageing. At the same time, cognitively healthy adults may have sub-clinical levels of AD pathology. As a result, ageing and AD may interact at the individual level, highlighting a methodological limit in the use of group comparisons.

To detect distinctive associations between the DMN and each of the two processes (ageing and AD), therefore, an alternative method based on linear regression was implemented. DMN functional connectivity was modelled as a function of: (1) a variable conceptually more linked to ageing than AD (age); and (2) a variable conceptually more linked to AD neurodegeneration than ageing (focal volume of the hippocampus). The statistical effects of these two predictors were modelled based on the increase in the fit of the model at the inferential level. Additionally, data were also analysed with canonical group comparisons, in order to highlight the different outcomes emerging from the use of distinct inferential methods. We expected that a pattern of statistical associations more specific to the neurodegeneration seen in AD than the outcome of group comparisons would emerge from these analyses.

## Material and Methods

### Participants

Three-hundred-and-seventy MRI datasets were considered for inclusion. This cohort was recruited between June 2011 and July 2016 in the memory clinic at the IRCCS Fondazione Ospedale San Camillo, in Venice, Italy as part of a project funded by the Italian Ministry of Health led by AV, and includes healthy adults aged between 22 and 85 years old, patients diagnosed with mild cognitive impairment^19^, and patients diagnosed with dementia of the AD type^20^. All diagnoses were reached by consensus among clinicians, and were based on a neurological screening, an extensive battery of neuropsychological tests, and, in a proportion of cases, follow-up appointments. A series of exclusion criteria served to rule out non-neurodegenerative aetiologies that might be behind the onset of cognitive symptoms (i.e., psychiatric, metabolic, traumatic or vascular). These criteria were defined as follows: evidence of a significant diagnostic entity, as revealed by MRI, which might account for the presence of cognitive impairment, depressive, anxious or other psychiatric symptoms of clinical relevance, pharmacological treatments with psychotropic medications, with drugs for research purposes or with toxic effects to internal organs, clinically significant diseases other than those consistent with the objective of the study, a previous history of transient ischaemic attacks, a diagnosis of severe vascular pathology with excessive hyperintensity load (quantified with a Fazekas score > 2)^21^, presence/diagnosis of uncontrolled seizures, peptic ulcer, sick sinus syndrome, neuropathy with conduction difficulties, significant disabilities, evidence of abnormal baseline levels of folates, vitamin B12 or thyroid-stimulating hormone. Following the application of these criteria, 34 datasets were excluded from the study. Moreover, 7 additional datasets were excluded as clinical history was incomplete and diagnosis uncertain. The remaining 329 MRI datasets were taken forward to the functional MRI (fMRI) preprocessing pipelines and were subjected to a quality check to rule out the presence of technical exclusion criteria. During these operations, 10 datasets were excluded because of signal artefacts affecting the BOLD signal, and 7 datasets were excluded because of excessive motion (see below for details). The final sample included 312 datasets (Table 1).

**Table 1:** Schematic description of the cohort.

Group	Number
I. Healthy Adults aged 21–40 years old	49
II. Healthy Adults aged 41–64 (*) years old	49
III. Healthy Adults aged 65–72 (**) years old	50
IV. Healthy Adults aged 73 or more	43
V. MCI patients with MMSE score > 27	39
VI. MCI patients with MMSE score 24–27	47
VII. Patients with AD Dementia (MMSE < 24)	35

(*) The separation bar was set at the age of 64 because adults younger than 65 who are diagnosed with AD are referred to as “early-onset” patients.

(**) The separation bar was set at the age of 72 because 72.8 years is the healthy life expectancy at birth in Italy, as estimated by the World Health Organization in 2015.

All participants completed an MRI protocol inclusive of T1-weighted and a resting-state fMRI acquisitions, plus a number of clinical sequences (diffusion-weighted, T2-weighted, and FLAIR), which were reviewed by a senior neuroradiologist to comply with study criteria, as described above. All participants aged 40 years old or older completed, as part of their clinical profiling, an extensive battery of neuropsychological tests to ascertain their clinical status. These included tests of short-term and working memory, episodic memory, lexical-semantic processing, abstract-conceptual reasoning, attentive-executive functions and visuoconstructive abilities. A complete description of the battery can be openly consulted in a previously published article^22^. Cognitive scores were made available for contingent *post hoc* analyses.

### Choice of the predictors for the study of ageing and AD

Two variables were chosen based on their association with the two developmental trajectories investigated in this study (ageing and AD). Although no demographic, pathological or clinical variable exists that truly depends on the expression of exclusively one of the two trajectories (i.e., a variable solely linked to ageing, or solely linked to AD), the following two indices emerged as having strong conceptual links “preferentially” with one of the two trajectories.

The “number of days of life at scan” was chosen as a variable more dependent on ageing than AD. This variable is equivalent to and more precise than the typical measurement in years. The “datedif” function in Microsoft Excel was used to calculate the exact number comparing the date of birth and the date of the MRI scan.

Hippocampal volume was instead chosen as a variable more dependent on AD than ageing. The hippocampus is one of the earliest areas affected in AD^23^ and is central in the typical presentation of the disease. Evidence of volumetric decrement of this structure is considered an important marker of AD-related neuronal injury^24^. Moreover, this variable is also informative among healthy adults, as hippocampal volume and integrity are predictors of cognitive decline in this population^25^. Finally, this variable offers a major methodological advantage over other potential proxies of AD (e.g., the score on the Mini Mental State Examination), because it is characterised by an ample numerical variability among old as well as young adults^26^, and, specifically in this latter sub-population, it is associated with memory performance^27,28^. Moreover, even at an age as young as ≈20 years, genetic variables that are biological modulators of the pathophysiological mechanisms of AD do appear to have an impact on the morphometry of the hippocampus^29,30^.

### MRI acquisition

Three-dimensional T1-weighted images and resting-state acquisitions were modelled for statistical inference. These sequences were acquired with a Philips Achieva 1.5 T machine as part of a single MRI protocol. Turbo Field Echo T1 images were based on a 1.1 × 1.1 × 0.6 mm^3^ (gap 0.6 mm), voxel resolution, 256 × 256 × 124 matrix size, 250 mm field of view, 7.4 ms repetition time, 3.4 ms echo delay time, and 8° flip angle. Resting state fMRI scans were preceded by 20 s of dummy volumes set to allow the scanner to reach equilibrium. At least 200 volumes were acquired for each participant, each volume consisting of 20 slices acquired axially and contiguously, in ascending order, with the following parameters: 3.28 × 3.28 × 6.00 mm^3^ voxel dimension, 64 × 64 matrix size, 230 mm field of view, 2 s TR, 50 ms TE and 90° flip angle.

### MRI processing

The methodology was carried out using Statistical Parametric Mapping software (SPM) 8 (Wellcome Centre for Human Neuroimaging, London, UK) and Matlab R2011b (Mathworks Inc., UK).

The SPM “new segmentation” tool was used to separate each T1-weighted image into six tissue maps. Of these, the two maps of neural tissue (grey matter and white matter) and the map of cerebrospinal fluid were quantified in volumetric terms using the “*get_totals*” script (www0.cs.ucl.ac.uk/staff/g.ridgway/vbm/get_totals.m). Total intracranial volumes represented the arithmetic summation of the volumetric quantification of the three maps for each participant. The grey-matter ratio was computed by dividing the total grey-matter volume by the total intracranial volume.

The hippocampus was processed using the STEPS procedure, fully automatised and available online (http://cmictig.cs.ucl.ac.uk/niftyweb/). This methodology allows an accurate segmentation of the hippocampus in its native space from T1-weighted images through the exploitation of multiple templates^31^. In order to minimise any effect of lateralisation, the volumes of the left and right hippocampus were averaged.

Resting-state fMRI scans were preprocessed using a standard pipeline that included slice timing, realignment, normalisation, temporal filtering (0.008–0.1 Hz) and a 6-mm smoothing. In-scanner motion parameters were inspected to verify the absence of excessive movement, which could induce artefactual alterations to the fluctuations of the blood oxygen level dependent signal. None of the scans had to show motion larger than the size of 1 voxel in any of the directions. Where excessive movements were located at the very beginning or very end of the functional run, the problematic volumes were removed (and, by doing so, the rhythmicity of neurogenic signal fluctuations was not altered), and the preprocessing pipeline was restarted. This was done for 6 scans, for which at least 180 volumes were retained. When excessive movement was instead located in the middle of the run, the participant was excluded from the study (7 datasets in total, as mentioned above).

A group independent component analysis was set up to extract the DMN^32^. To do so the GIFT toolbox was used (GIFT v1.3i; mialab.mrn.org/software/gift). The Infomax optimisation principle was chosen, and the number of components was set at 20, following the choice made in a very large study of more than one thousand scans^33^. Given the very high inter-rater level of agreement on the identification of the DMN among the output components^34^, the map with the spatial characteristics of the DMN was selected based on the judgement of two independent raters.

### MRI modelling

Descriptive statistics were run to characterise the distribution of the two proxies, and Pearson’s *r* coefficients of correlation were calculated to test for collinearity.

Linear models were devised to predict the variability of DMN connectivity as expressed by *z* scores. In order to replicate the established finding that ageing and AD have an effect on the connectivity of the posterior section of the DMN, *t* tests were devised. A first model was run to compare a sub-group of patients with clinically-established dementia of the AD type (group VII in Table 1) and a sub-group of healthy adults matched for age (*p* = 0.457), education (*p* = 0.471) and gender (*p* = 0.878). A second model was then run to compare a sub-group of young adults (group I in Table 1) and a sub-group of healthy elderly adults (group IV in Table 1). These two group comparisons were run to test for differences ascribable to AD and ageing on the DMN, respectively.

To explore the distinctive predictive effect of the two predictors, uncorrected multiple regression models were initially set up. Subsequently, multiple regression models were run correcting for the homologous proxy (i.e., the predictive effect of age correcting for hippocampal volume, and the predictive effect of hippocampal volume correcting for age). Finally, fully-corrected multiple regression models were run, including the homologous proxy, gender, levels of education (to control for cognitive reserve), total volumes of grey matter (to control for brain reserve), and the ratio of grey matter (to control for global atrophy). These two latter covariates were not correlated with one another (*r* = 0.055, *p* = 0.335). All contrasts were devised in the direction of our hypotheses (i.e., a negative association between network connectivity and age, and positive association between network connectivity and hippocampal volume). The same inferential models were also run to predict the effect of age and hippocampal volume on the whole-brain map of grey matter.

### Ethical approval

All procedures involved in this study were carried out in accordance with institutional ethical standards and with the 1964 Helsinki declaration and its later amendments. This study had received approval by the Institutional Review Board of the IRCCS Fondazione Ospedale San Camillo (Venice, Italy), (Protocol No. 11/09 version 2).

### Informed consent

Informed consent was obtained from all individual participants included in the study.

### Sharing of data

The authors have no permission from participants to share their data beyond the team of collaborators of the principal investigator (AV).

## Results

**Figure 1:**
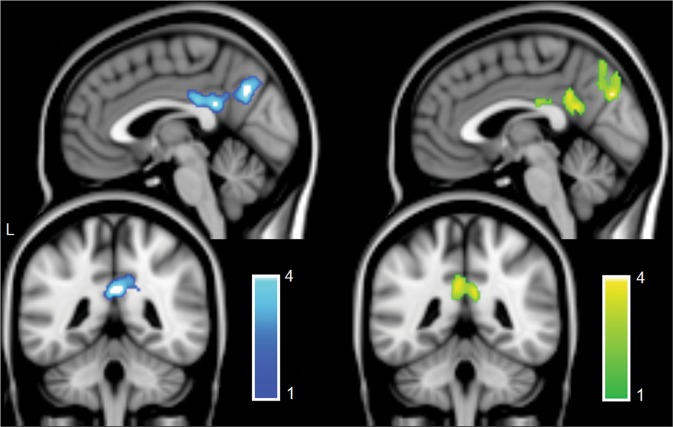
Results of the *t* test models testing the effect of ageing (healthy young > healthy old, cyan overlay) and the effect of AD (healthy old > AD dementia, green overlay) on the DMN. The same pattern emerged from the two models (x = −2; y = −44). *z* scores are indicated on the side of each output.

The correlation between the two predictors was significant but was limited to *r* = −0.326, ruling out collinearity issues. The results of the two *t* tests run on sub-groups of the cohort are reported in Fig. 1 and Table 2. Healthy elderly adults had more connectivity than patients with AD dementia in a large postero-medial cluster encompassing Brodmann Area (BA) 7, 23 and 31 (posterior cingulate/retrosplenial cortex and precuneus). Similarly, young adults had more connectivity than elderly adults in the same areas.

**Table 2:** Effect of Alzheimer’s disease degeneration and of the process of ageing on the functional connectivity of the DMN detected by between-sample *t* test models.

Cluster No.	Cluster *pFWE*	Cluster size (voxels)	*Z* Score at Local Maximum	Side	BA	Region	Talairach Coordinates		
							x	y	z
***Healthy Elderly Adults*** > ***AD Patients***									
1	0.000	1640	4.85	L	7	Cuneus	−6	−72	33
			4.28	R	7	Cuneus	12	−70	33
			4.19	L	7	Cuneus	−12	−68	29
			3.72	L	31	Cingulate Gyrus	−4	−43	30
			3.70	R	7	Precuneus	10	−60	36
			3.68	L	31	Cingulate Gyrus	−10	−29	33
			3.65	L	23	Posterior Cingulate	−4	−47	23
			3.64	R	7	Precuneus	2	−65	51
***Young Adults*** > ***Healthy Elderly Adults***									
1	0.000	1290	4.82	R	31	Cingulate Gyrus	4	−41	26
			4.73	R	23	Cingulate Gyrus	6	−20	27
			4.47	L	7	Precuneus	−2	−62	36
			3.96	R	31	Precuneus	14	−47	34
			3.78	L	23	Cingulate Gyrus	−2	−28	27
			3.54	L	31	Precuneus	−10	−55	30
2	0.003	522	4.31	R		Claustrum	36	14	1
			3.99	R	13	Insula	38	10	−2

BA: Brodmann Area; L: Left; R: Right.

**Figure 2:**
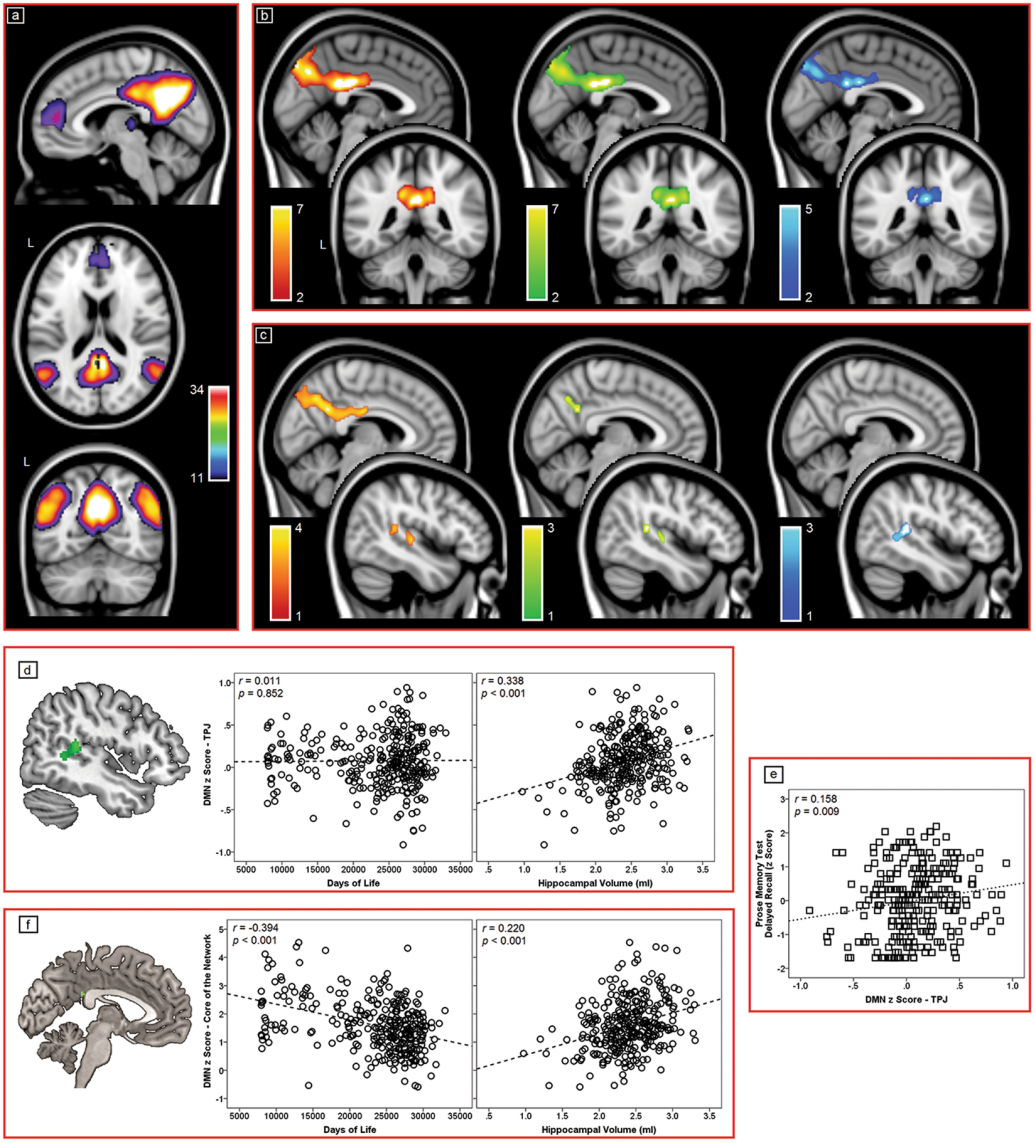
(**a**) The DMN map, as estimated with a one-sample *t* test carried out on the entire cohort and controlling for both proxies and all covariates (x = 6; z = 18; y = −62), and linear regression models testing the association between each of the proxies and functional connectivity of the DMN. Specifically, (**b**) the negative association between functional connectivity and the ageing proxy (x = −2; y = −44), and (**c**) the positive association between functional connectivity and the AD proxy (x = 6; x = 46). Uncorrected associations are shown in red, models corrected for the homologous proxy are shown in green, and the fully-corrected models are shown in cyan. *z* scores are indicated on the side of each output. (**d**) Association between functional connectivity of the DMN within the right TPJ (expressed as an average of *z* scores) and each of the two main independent variables of this study. (**e**) Association between the DMN signal in the right TPJ and performance on the Prose Memory test (delayed recall). Finally (**f**), the association between functional connectivity of each of the two proxies and the main DMN core. This core regions was located in the posterior cingulate cortex (BA 31, *p*FWE = 0.001, cluster extent: 12 contiguous voxels, peak Talairach coordinate: x = −4, y = −38, z = 26). The dotted lines represent linear associations. Pearson’s *r* coefficients and respective *p* values are shown. DMN: Default-Mode Network; TPJ: Temporo-Parietal Junction.

The results of the regression models investigating the pattern of distinctiveness for each predictor are illustrated in Fig. 2. A negative association was found between age and functional connectivity of the DMN in a large postero-medial region covering the retrosplenial/posterior cingulate cortex and the precuneus, and in the insula bilaterally. This was partially mitigated after controlling for hippocampal volumes, and still retained its core features in the fully-corrected model (Table 3, Fig. 2b). The uncorrected model revealed a similar (positive) association between hippocampal volumes and DMN connectivity of a large postero-medial region, and a smaller cluster located at the border of the right temporal and parietal lobe (showing its peak in BA 22 but extending also to BA 21, 40, 41, and 42). In the partially-corrected model the postero-medial cluster was largely downsized, and it was no longer significant in the fully-corrected model. On the other hand, the right temporo-parietal cluster retained significance (Fig. 2c). Using the left or right hippocampal volume as proxy instead of the bilateral average resulted in the same pattern of results. Similarly, the inclusion of total intracranial volumes among the covariates (in lieu of the ratio of grey matter) resulted in unaltered findings.

**Table 3:** Distinctive and exclusive association between functional connectivity of the DMN and age/hippocampal volume.

Cluster Number	Cluster pFWE	Cluster size (voxels)	Z Score at Local Maximum	Side	BA	Region	Talairach Coordinates		
							x	y	z
***Negative association between age and DMN***									
1	0.000	2443	6.05	R	31	Cingulate Gyrus	2	−37	28
			6.00	R	7	Precuneus	2	−66	38
			5.45	L	23	Cingulate Gyrus	−2	−26	27
			4.97	L	7	Precuneus	−2	−75	44
			4.62	R	7	Precuneus	10	−75	50
			4.13	L	31	Precuneus	−12	−57	29
			4.10	L	31	Precuneus	−6	−49	30
			3.77	R	39	Middle Temporal Gyrus	30	−53	27
			3.76	L	31	Precuneus	−10	−45	34
2	0.043	351	3.74	R	13	Insula	42	−13	4
			3.63	R	41	Transverse Temporal Gyrus	53	−15	8
***Positive association between hippocampal volume and DMN***									
1	0.002	569	4.37	R	41	Transverse Temporal Gyrus	34	−36	15
			4.04	R	41	Superior Temporal Gyrus	50	−34	13
			3.87	R	41	Superior Temporal Gyrus	57	−29	11

BA: Brodmann Area; L: Left; R: Right.

To characterise the role of this temporo-parietal region more in detail, the local DMN signal was extracted. Correlation models were created to test its association with the two independent variables and performance on all cognitive tests. The association between the strength of the DMN in this cluster (expressed as *z* scores) and the two independent variables is illustrated in Fig. 2d. This value was only associated with hippocampal volume, not with age. Moreover, the sole neuropsychological test showing a significant association with regional DMN strength was the recall of the Prose Memory test (*r* = 0.158, *p* = 0.009; Fig. 2e). This association was even more significant after correcting for age and for all covariates included in the main models, and the DMN connectivity in the posterior cingulate cortex (*r* = 0.180; *p* = 0.003).

## Discussion

Evidence indicates that the posterior portion of the DMN seems to be particularly susceptible to both the process of normal ageing and the pathological impact of AD. With a first set of analyses, we confirmed these well-established findings: ageing and AD are both associated with decreased functional connectivity of the DMN in the posterior cingulate/retrosplenial cortex and precuneus. These results were obtained with *t* tests comparing groups of participants. A different pattern, however, emerged from the regression models. In these models we focused on the distinctive impact of two variables known to be conceptually more linked either with normal ageing (days of life at scan) or neurodegeneration of the AD type (hippocampal volume). The results indicate that the association between functional connectivity in the posterior cingulate/retrosplenial cortex and precuneus and days of life at scan was significant beyond the statistical prediction offered by hippocampal volume. This does not mean that hippocampal volume does not predict functional connectivity in this region, or in this specific cohort (in fact, we did replicate with t test models the findings reported in the literature), but it means that the numerical variability predicted by hippocampal volume in the postero-medial territory is not significantly more than that predicted by age. This indicates that the typical posterior reductions of functional connectivity seen in the DMN do not seem to be a specific marker of abnormal ageing, but rather represent a general vulnerability of brain physiology to multiple processes, as already proposed^35^.

A novel finding of this study is the exclusive predictive impact of hippocampal volumes on the DMN functional connectivity in a cluster located in the right hemisphere and covering part of the posterior temporal and inferior parietal lobes. This region is part of the anterior portion of the temporo-parietal junction (TPJ) territory^36^. The right TPJ (rTPJ) is an associative area involved in theory-of-mind processes^37,38^, and in the conceptually similar construct of “mentalizing”^39^. As social-cognition skills, these abilities rely on “a network of areas at least partly overlapping with the DMN”^40^, and the DMN itself is crucial for sustaining a skill like theory of mind^41^. It is, therefore, expected that reduced DMN strength in the rTPJ may lead to reduced abilities of social cognition in the AD trajectory. Unfortunately, no formal measure of social cognition was available for the participants included in this study. However, it is particularly significant that the strength of the DMN in the rTPJ was associated with performance on the recall part of the Prose Memory test with a *p* < 0.01. The short stories used to test verbal episodic learning (such as the logical-memory test of the Wechsler Memory Scale, or the Babcock story) are usually structured around events that are prone to elicit a strong empathic response (i.e., the robbery and parenting issues Anna Thompson has difficulties with, or the tragedy that strikes the victims of the flooding river). Similarly, theory of mind and TPJ function are tested via the administration of short stories that are conceptually equivalent^42^. Moreover, memory and theory-of-mind abilities share neuroanatomical correlates^43,44^ and a tight bi-directional interplay exists between the two functions: more details about somebody’s life story are remembered, more vivid is the use of theory-of-mind skills in that specific context^45^. Conversely, processing information via a social cognitive route improves encoding and memory performance^46^. Within this context, it is not a coincidence that recent evidence also shows that when encoding of information has a social connotation, it engages the rTPJ^47^. In summary, although social cognition abilities were not directly investigated in the present study, converging evidence indicates, beyond simple speculation, that a conceptual thread connects rTPJ, social cognition and memory, and that the *post hoc* association we found between memory performance and the strength of the DMN in the rTPJ may pave the way for the study of social cognition as a domain that could be discriminatory between normal ageing and AD. It is known that AD patients show a profound impairment in social cognition, which is independent of the impairment in general cognition^48^. Vice versa, healthy elderly adults show no decrement in theory-of-mind skills compared to young adults^49^. Moreover, difficulties in perspective taking contribute to increased anosognosia. A study of cortical metabolism found that the reduction of glucose uptake in the TPJ territory was associated with poorer disease awareness^50^. A solid link between anosognosia and DMN function exists. Autobiographical memory, envisioning of the future, and theory of mind are three examples of “DMN task” in which the personal perspective has to shift, and a “self-projection” is requested^1^. Along these lines, anosognosia can be considered, for all intents and purposes, a failure of self-projection. Based on this, loss of functional connectivity between the TPJ and computational hubs of the DMN will result into a disconnection between the ability to adopt a certain viewpoint during mentation and awareness of cognitive deficits, with an incidental effect on memory abilities when these are tested with tasks rich in empathy-inducing content.

Although the TPJ is not usually considered a “prototypical” area distinctively affected by AD pathology (like the transenthorinal region, the hippocampus, or the posterior cingulate cortex), recent studies have described in explicit terms the TPJ as a central region significantly affected by AD pathological changes. Together with the posterior hippocampus and the posterior cingulate cortex, the “temporo-parietal junction seem to make an important contribution in the longitudinal progression during the very early stages of amyloid-β accumulation”^51^^, page2249^. Similarly, the TPJ is (together with the temporal pole) the region with the most elevated uptake of TAU binding tracer^52^, and is characterised by reduced glucose metabolism both in early-onset and late-onset AD^53^. Altogether, these findings are highly convergent towards a link between alterations seen in the TPJ and the spectrum of pathological and neurofunctional alterations seen in AD.

This study is not free from limitations. On one hand, the choice of days of life at scan and hippocampal volumes as predictors offered important advantages. These include construct validity (i.e., the presence of a net theoretical association between each predictor and the construct the predictor is meant to quantify), excellent numerical variability and absence of floor or ceiling effects. However, other variables could be selected as indices more linked to physiological ageing or neurodegeneration (e.g., an index of cellular ageing such as telomere length, rather than “demographic ageing”, and a measure of regional brain metabolism instead of brain morphology). Although we acknowledge our choices as theoretically sound and methodologically strong, we cannot rule out the possibility that other selections could have yielded small differences in the pattern of findings. Second, the idea of “independence” operationalised by our methodology was bound to to the concept of significant improvement in the model fit. No measurable variable exists that is exclusively associated with ageing (and not AD) or exclusively associated with AD (and not ageing). Statistics, however, offers a valuable method to study the prediction offered by a variable after partialling out the amount of variability predicted by a second variable of no interest and calculating thus its distinctive contribution to the model. Third, we identified the DMN as a single independent component based on haemodynamic regularities. Other studies separated the DMN into two or more subsystems^17,54,55^, often labelled anterior DMN and posterior DMN. We analysed one pattern of connectivity only because we found only one map which showed the typical topography characteristics of the DMN.

## Conclusion

In summary, although group-comparison models indicate that ageing and AD are associated with the statistical strength of activity in the DMN in a qualitatively similar manner, regression models provided statistical evidence indicating that the typical pattern of associations seen in postero-medial regions appears not to be a distinctive sign of neurodegeneration. Hippocampal volumes were instead predictive of DMN connectivity within the rTPJ. The role of this structure in social cognition and awareness of disease suggests the study of these symptoms deserves more attention for the development of a clinical marker able to detect sporadic AD in its early stage.
